# How to Define Biomarkers of Human T Cell Aging and Immunocompetence?

**DOI:** 10.3389/fimmu.2013.00136

**Published:** 2013-06-04

**Authors:** Dietmar Herndler-Brandstetter, Harumichi Ishigame, Richard A. Flavell

**Affiliations:** ^1^Department of Immunobiology, Yale University School of MedicineNew Haven, CT, USA; ^2^Howard Hughes Medical Institute, Yale University School of MedicineNew Haven, CT, USA

In 2012, 13–22% of the population in Europe, the US, and China were older than 60 years of age. In 2050, demographic models predict that 27–34% of the population in these countries will be older than 60 years of age. Worldwide, more than two billion persons will be age 60 years or over by 2050 (United Nations Department of Economic and Social Affairs Population Division, 2012). This will pose an enormous medical and socioeconomic challenge to our future society. One of the most recognized consequences of aging is a decline in immune function, which limits the protective effect of vaccinations and renders elderly people more susceptible to certain infectious diseases and to newly emerging infections, such as influenza (Gavazzi and Krause, 2002; Goronzy and Weyand, 2013). A higher prevalence of urinary tract infections, lower respiratory tract infections, skin and soft tissue infections, infective endocarditis, bacterial meningitis, tuberculosis, and herpes zoster has been observed in the elderly. Yet, infections in the elderly are not only more frequent but also more severe and have distinct features with respect to clinical presentation (Gavazzi and Krause, 2002). Accordingly, pneumonia, influenza, and septicemia are ranked among the 10 major causes of deaths in people aged 65 years and over in developed countries.

Although considerable progress has been made to identify the cellular changes and molecular mechanisms underlying T cell aging, we still lack biomarkers of T cell aging that have been validated in large populations and that correlate with functional immune responsiveness. This opinion article will therefore focus on how aging affects the number, phenotype, and function of human naïve and memory T cells, and how to identify and validate potential biomarkers of T cell aging. The availability of a valid set of biomarkers will be of utmost importance to improve medical and preventive treatments in the elderly and to evaluate potential therapies that aim to rejuvenate the aged immune system.

## The Impact of Aging on Human Naïve T Cells

Of the many different types of immune cells, the decline of T cell number and function appears to be a key feature of human immune cell aging. This is due to a decreased capacity of aged hematopoietic stem cells to generate committed lymphoid progenitors and an age-related atrophy of the maturation organ for T cells, the thymus (Linton and Dorshkind, 2004; Geiger et al., 2013). At the age of 50 years, 90% of functional thymic tissue has been lost. This leads to a dramatic shortage of naïve T cells in the peripheral blood (PB), lymph nodes, and bone marrow (BM) (Fagnoni et al., 2000; Lazuardi et al., 2005; Herndler-Brandstetter et al., 2012). Further evidence that supports the key role of the thymus to maintain naïve T cell number and function derives from a study demonstrating premature T cell aging in patients thymectomized during early childhood (Sauce et al., 2009). A decline of naïve T cells in the PB and BM has also been observed in individuals with persistent viral infections, in particular with human cytomegalovirus (HCMV) infection (Almanzar et al., 2005; Herndler-Brandstetter et al., 2012). This indicates that the expansion and accumulation of high numbers of HCMV-specific effector-memory T cells (T_EM_) leads to an exhaustion of the naïve T cell pool (Sylwester et al., 2005). Another possibility could be that HCMV infection itself contributes to thymic atrophy, as has been reported for murine CMV (MCMV) infection (Price et al., 1993). In summary, the rate of naïve T cell decline during aging is determined by two key factors: the pace of thymic atrophy and certain persistent infections.

The aging process also changes the *quality* of naïve T cells. There are two subsets of naïve (CD62L^+^CD45RA^+^) CD4^+^ T cells in the PB of humans, defined by the presence or absence of CD31 (PECAM-1) (Kimmig et al., 2002). CD31^+^ naïve CD4^+^ T cells may resemble recent thymic emigrants, with a high content of T cell receptor excision circles (TRECs), while CD31^−^ naïve CD4^+^ T cells have a lower TREC content and a restricted T cell repertoire (TCR). The loss of CD31 expression has been attributed to TCR-mediated peripheral post-thymic homeostatic proliferation. Accordingly, CD31^+^ naïve CD4^+^ T cell numbers decline during aging in the PB. Yet, aged naïve CD4^+^ T cells respond with an unimpaired IL-2 production upon stimulation with neoantigen and major signaling defects, such as the lack of calcium influx observed in aged mouse naïve T cells, are not seen in humans (Gomez et al., 2004; Tsukamoto et al., 2009). However, human naïve CD4^+^ T cells from elderly individuals have a reduced TCR-mediated signaling capacity of the ERK pathway due to an age-related decline in miR-181a (Li et al., 2012). Although CD31^+^ naïve CD4^+^ T cells were already described in 2002, no follow-up studies have validated CD31 as a functional biomarker of immunocompetence, e.g., protection after immunizations with neoantigens in elderly persons or decreased risk of influenza-associated hospitalization.

Similar to human naïve CD4^+^ T cells, aged human naïve (CD62L^+^CD45RA^+^) CD8^+^ T cells display a dramatically restricted TCR repertoire, shortened telomeres and express IL-6Rα and IL-7Rα at a lower frequency (Pfister et al., 2006; Alves et al., 2007; Herndler-Brandstetter et al., 2011). The narrowing of the TCR repertoire by homeostatic proliferation may be explained by the preferential selection of naïve T cells for high TCR:pMHC avidity, as recently shown in a mouse model. This high avidity naïve CD8^+^ T cells underwent faster rates of homeostatic proliferation and preferentially acquired a “memory-like” phenotype (Rudd et al., 2011). This study accentuates an important problem that has not received great attention. How reliable are surface markers, such as CD62L, CD45RA, CCR7, CD27, and CD28 in identifying aged human naïve T cells? Not only are memory T cells able to re-express CD45RA and CCR7 (Wills et al., 1999; van Leeuwen et al., 2005), but, and more importantly, naive T cells that undergo extended homeostatic proliferation acquire a memory-like phenotype. This has been demonstrated for murine naïve CD8^+^ T cells (Murali-Krishna and Ahmed, 2000). In humans, data are very limited. However, a non-regulatory CD62L^+^CD45RO^+^CD25^dim^ CD8^+^ T cell population has been identified in healthy, HCMV-seronegative elderly persons who characteristically still had a good humoral response following influenza vaccination (Schwaiger et al., 2003; Herndler-Brandstetter et al., 2005). This novel memory-like CD8^+^ T cell population had a relatively diverse TCR repertoire, long telomeres, and produced large amounts of IL-2, and may therefore encompass homeostatically expanded naïve T cells (Herndler-Brandstetter et al., 2008).

In conclusion, naïve T cell numbers decline during aging, aged naïve T cells have a restricted TCR diversity and shortened telomeres but seem to retain some of their functional properties. Yet, naïve T cell numbers may be underestimated, as life-long homeostatic proliferation of aged naïve T cells may display a memory-like phenotype. Unfortunately, no large-scale studies have evaluated whether high numbers of naïve T cells with a diverse TCR repertoire and intact IL-2 production correlate with an intact immune responsiveness following vaccination with neoantigens in elderly persons or whether such elderly individuals have a decreased risk of influenza-associated hospitalization.

## The Impact of Aging on Human Memory T Cells

The ability to generate and maintain functional memory T cells following infection or vaccination is a hallmark of the adaptive immune system and ensures protection upon recurrent infections. In old mice, the generation of functional CD4^+^ and CD8^+^ T cell memory is impaired, which has been attributed to functional defects in naïve T cell stimulation and decreased effector T cell expansion (Kapasi et al., 2002; Haynes et al., 2003). In addition, the aged murine microenvironment, in particular defects in T cell migration, priming by antigen presenting cells and differentiation into follicular T helper cells, contributes to a suboptimal CD4^+^ T cell-mediated immune response.

In humans, memory T cells seem to be less affected by the aging process compared to naïve T cells. For example, CMV-specific T cell immunity is maintained in immunosenescent rhesus macaques and overt CMV disease is rare in the elderly (Rafailidis et al., 2008; Cicin-Sain et al., 2011). However, herpes zoster, which is caused by reactivation of the Varicella zoster virus that causes chickenpox in children, occurs more frequently in the elderly. Following routine vaccinations in healthy and frail elderly persons, decreased IgG antibody concentrations, delayed peak antibody titers, and a more rapid decline in antibody titers were observed compared to young adults (Weinberger et al., 2008). A decreased tumor necrosis factor (TNF)-α synthesis by macrophages also restricts cutaneous immunosurveillance by memory CD4^+^ T cells during aging, which may thereby contribute to the increased susceptibility to cutaneous infections and malignancies in older humans (Agius et al., 2009). In summary, these studies indicate that memory T cells, as well as their interaction with B cells and antigen presenting cells, are less efficient in old age.

In humans, three major classes of memory T cells can be distinguished based on their phenotypic and functional characteristics: central-memory T cells (T_CM_) with a CD45RO^+^CD28^+^CD62L^+^ phenotype, T_EM_ with a CD45RO^+^CD28^+^CD62L^−^ phenotype, and highly differentiated T_EM_ (CD28^−^ T cells) with a CD45RO^±^CD28^−^CD62L^−^ phenotype. During human aging, the number of T_EM_ and CD28^−^ T cells increases in the PB and BM (Almanzar et al., 2005; Kovaiou et al., 2005; Herndler-Brandstetter et al., 2012). The accumulation of effector-memory CD28^−^CD8^+^ T cells, which have a highly restricted TCR repertoire, shortened telomeres, and decreased antigen-induced proliferation, has been included in a set of parameters defining the immune risk phenotype and correlates with a lack of antibody production after influenza vaccination in elderly persons (Olsson et al., 2000; Saurwein-Teissl et al., 2002). The loss of the co-stimulatory molecule CD28 and the consequent age-dependent accumulation of CD28^−^CD8^+^ T cells can be attributed to two mechanisms: repeated antigenic stimulation and IL-15-mediated homeostatic proliferation (Valenzuela and Effros, 2002; Almanzar et al., 2005; Chiu et al., 2006). Although there is some confusion in the literature about how to accurately describe this CD28^−^CD8^+^ T cell population, the term “highly differentiated” may be most suitable. Other descriptions, such as dysfunctional or senescent are misleading and do not reflect the properties of CD28^−^CD8^+^ T cells, as these cells are not anergic, are susceptible to apoptotic cell death, proliferate upon proper stimulation and are highly cytotoxic (Chiu et al., 2006; Waller et al., 2007; Brunner et al., 2012). As large numbers of highly differentiated CD28^−^CD8^+^ T cells accumulate during aging, the targeted depletion of these cells has been proposed to generate more “space” for naïve and T_CM_ survival. However, CD28^−^CD8^+^ T cells may be important for tissue-mediated immunity and due to their lack of lymph node homing markers, these cells are likely to occupy different niches than naïve and T_CM_ (Remmerswaal et al., 2012). Moreover, a sudden drop of T cell numbers due to depletion of CD28^−^CD8^+^ T cells may lead to massive peripheral naïve T cell proliferation.

In conclusion, although several human memory T cell subsets have been defined, we still lack information about their origin and maintenance, their functional and migratory properties. The identification of microenvironmental niches of specific memory T cell subsets, in particular of CD28^−^ T cells, would enable the search of novel markers to distinguish homeostasis- from repeated antigen-driven memory T cells.

## Concluding Remarks

Age-related changes within the human T cell pool have almost exclusively been studied in cells derived from the PB. However, the PB contains only two percent of the total body T cell pool (Di Rosa and Pabst, 2005). Very limited data are available how aging affects naïve and memory T cells in lymphoid and extra-lymphoid organs (Lazuardi et al., 2005; Herndler-Brandstetter et al., 2012; Sathaliyawala et al., 2013). In particular, as memory T cells in non-lymphoid tissues have been shown to provide enhanced local immunity during infection (Gebhardt et al., 2009). A prerequisite for defining biomarkers of human immune aging and testing strategies to reverse or delay immunological aging is to analyze naïve and memory T cell populations in different organs and validate their phenotypic and functional characteristics. The impact of aging on T cells in distinct microenvironmental niches would also help us to, e.g., identify phenotypic and functionally distinct subpopulations of CD28^−^CD8^+^ T cells that may have been generated by either chronic antigenic stimulation or life-long homeostatic proliferation. The functional analysis of aged human naïve and memory T cell subsets *in vivo*, e.g., in humanized mice, may be another promising strategy to enhance our understanding about human T cell aging (Rongvaux et al., 2013). Large-scale integrated projects that aim to define biomarkers of aging, such as the EU-funded project MARK-AGE, are also underway and may pave the way for personalized medical treatment and preventive interventions in the elderly.
